# Predictors of clinically diagnosed premature ventricular complexes in acute care settings: a cohort study

**DOI:** 10.1093/europace/euag204

**Published:** 2026-08-04

**Authors:** Jean Jacques Noubiap, Gabrielle C Montenegro, Catherine Lee, Janet J Tang, Thomas A Dewland, Edward P Gerstenfeld, Gregory M Marcus

**Affiliations:** Division of Cardiology, Department of Medicine, University of California-San Francisco, 505 Parnassus Avenue, M1180B, San Francisco, CA 94143, USA; Division of Cardiology, Department of Medicine, University of California-San Francisco, 505 Parnassus Avenue, M1180B, San Francisco, CA 94143, USA; Department of Epidemiology and Biostatistics, University of California-San Francisco, San Francisco, CA, USA; Division of Cardiology, Department of Medicine, University of California-San Francisco, 505 Parnassus Avenue, M1180B, San Francisco, CA 94143, USA; Division of Cardiology, Department of Medicine, University of California-San Francisco, 505 Parnassus Avenue, M1180B, San Francisco, CA 94143, USA; Division of Cardiology, Department of Medicine, University of California-San Francisco, 505 Parnassus Avenue, M1180B, San Francisco, CA 94143, USA; Division of Cardiology, Department of Medicine, University of California-San Francisco, 505 Parnassus Avenue, M1180B, San Francisco, CA 94143, USA

**Keywords:** premature ventricular complexes, Predictors, Cohort

Premature ventricular complexes (PVCs) are common arrhythmias often detected on electrocardiography or ambulatory monitoring.^1,2^ Although traditionally considered benign, frequent PVCs are associated with heart failure, stroke, and mortality, and are increasingly recognized as clinically significant.^1^ Prior studies have identified associations with age, male sex, and cardiovascular comorbidities,^1–4^ but were limited by small or selected populations, short monitoring durations, and cross-sectional assessments. Consequently, population-level predictors of clinically diagnosed PVCs in acute care settings remain unclear. In parallel, therapeutic options for symptomatic or high-burden PVCs, particularly catheter ablation, have advanced considerably.^1,2^ Identifying factors associated with PVC diagnoses may help clarify which patients are most likely to present for care, inform risk stratification, and highlight opportunities for prevention. Hence, we performed a population-based, longitudinal cohort study to explore factors associated with clinically diagnosed PVCs in contemporary practice.

We used data from the California Department of Health Care Access and Information (HCAI) databases, including adults aged ≥18 years who had an encounter in ambulatory surgery units, emergency departments, or inpatient hospitals between 1 January 2016, and 31 December 2022. Patients were followed longitudinally to capture repeated encounters across care settings. Demographic variables included age, sex, race/ethnicity, insurance type, and income quartile. Race and ethnicity were harmonized into mutually exclusive categories. Comorbidities and substance use (including alcohol, tobacco, cannabis, cocaine, amphetamines, and opioids) were identified using the International Classification of Diseases, Tenth Revision, Clinical Modification (ICD-10-CM) codes and updated longitudinally, with conditions carried forward after diagnosis. The outcome of interest was incident clinically diagnosed PVC (excluding cases of PVC diagnosis at baseline). Multivariable Cox proportional hazards models were used to identify factors associated with PVC diagnosis. A *P*-value of < 0.05 was considered significant. Analyses were performed using the Stata 18 statistical package (Statacorp, College Station, TX). This study was approved by the IRB of the University of California-San Francisco.

Among 20 234 245 individuals, 153 005 incident cases of PVCs occurred over a median of 4.8 years. Patients with PVCs were older, more often male and White, and were more likely to use substances and to have comorbidities (all *P* < 0.001). In multivariable analyses, increasing age and male sex were associated with higher risk of PVC diagnosis (*Figure 1*). Race and ethnicity were significantly associated with PVC diagnoses. Compared with White individuals, Black individuals had a modestly higher risk, whereas Hispanic, Asian/Native Hawaiians or Pacific Islanders had lower risks, with no difference for American Indians or Alaska Natives. Higher-income individuals were more likely to have a PVC diagnosis.

**Figure 1:**
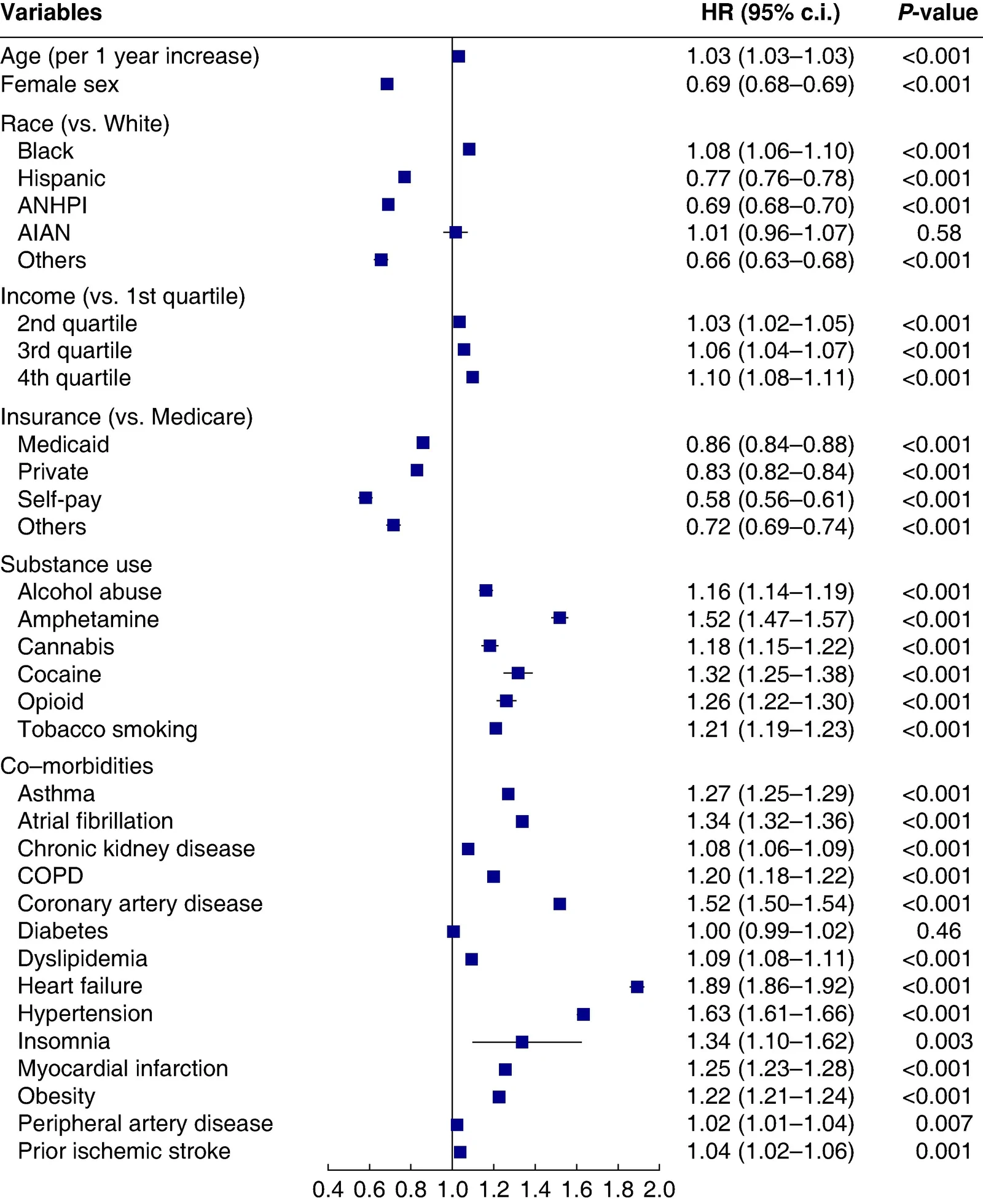
Predictors of premature ventricular complex diagnosis. 95% CI: 95% confidence interval; AIAN: American Indian or Alaska Native (AIAN); ANHPI: Native Hawaiian or Other Pacific Islander; COPD: chronic obstructive pulmonary disease; HR: hazard ratio; PVC: premature ventricular complex.

Among the substances studied, amphetamine use demonstrated the strongest association (hazard ratio [HR] 1.52), followed by cocaine (HR 1.32), opioids (HR 1.26), tobacco smoking (HR 1.21), cannabis (HR 1.18), and alcohol use (HR 1.16) (all *P* < 0.001). Cardiovascular comorbidities were among the strongest predictors of PVC diagnosis. Heart failure exhibited the strongest association (HR 1.89), followed by hypertension (HR 1.63), coronary artery disease (HR 1.52), atrial fibrillation (HR 1.34), and prior myocardial infarction (HR 1.25). Additional associations were observed for chronic kidney disease, dyslipidaemia, chronic obstructive pulmonary disease, asthma, obesity, stroke, peripheral artery disease, and insomnia. In contrast, diabetes mellitus was not significantly associated with PVC diagnosis.

While prior studies have largely relied on ambulatory monitoring or selected cohorts,^1–5^ our study captures predictors of PVCs that reach clinical attention and are documented during healthcare encounters. The strong associations with structural heart disease and cardiovascular comorbidities reinforce the concept that PVCs often reflect underlying myocardial substrate and disease burden.^1^ Differences observed across demographic and socioeconomic factors may reflect a combination of biological variation, differential exposure to risk factors, and disparities in healthcare access.

The observed associations with substance use are particularly notable. Stimulants such as amphetamines and cocaine may predispose to ventricular ectopy through ion channel dysfunction, QT prolongation, and electrical and structural remodelling leading to sympathetic activity and myocardial excitability.^6^ Cannabis may contribute to arrhythmogenesis through autonomic dysregulation and possible effects on myocardial ion channels and oxidative stress, although direct mechanistic evidence linking cannabis specifically to ventricular ectopy remains limited.^7^ Alcohol and tobacco smoking may also promote ventricular ectopy through sympathetic activation, oxidative stress, altered calcium handling and ion channel function, myocardial fibrosis, and direct cardiomyocyte toxicity, thereby increasing myocardial excitability and susceptibility to ventricular arrhythmias.^8,9^ The association of the use of these substances with diagnosed PVCs underscores the importance of screening for substance use in patients with PVCs and highlights potential targets for prevention.

Diabetes mellitus was not independently associated with clinically diagnosed PVCs after multivariable adjustment despite previous studies reporting positive associations.^1,3,4^ This likely reflects mediation of diabetes-related arrhythmic risk through downstream cardiovascular complications, including hypertension, coronary artery disease, heart failure, and chronic kidney disease, which were included in our model. It is interesting to consider the possibility that diabetic-associated neuropathy might reduce the subjective sensations produced by PVCs, leading to less healthcare utilization that might otherwise occur due to the diagnosis.

Our study has several limitations. Use of administrative data and diagnostic codes may introduce misclassification and incomplete capture of PVCs and comorbidities and precluded the ability to quantify relative frequencies of PVCs between individuals. Although prior work supports the validity of the diagnoses used based on ICD-10 codes,^10^ the diagnostic accuracy of the ICD-10-CM code for PVCs has not been specifically validated. Because we included only individuals presenting to healthcare settings, our study population may not be fully representative of the general population in California. Our findings reflect clinically recognized PVCs and may not generalize to asymptomatic or undetected cases. Residual and unmeasured confounding cannot be excluded. Finally, although based on a large and diverse California population, findings may not be fully generalizable to other settings or healthcare systems.

In conclusion, in a large, population-based cohort of over 20 million individuals, clinically diagnosed PVCs in acute care settings were strongly associated with older age, male sex, substance use, and cardiovascular comorbidities. These findings highlight key demographic and clinical factors associated with PVC diagnoses in contemporary practice and may inform risk stratification and preventive strategies.

## Authors’ contributions

Conception and design: GMM, JJN. Access to data: JJN, GMM, GCM, JJT, TAD. Data analysis and interpretation: JJN, GMM, CL. Manuscript drafting: JJN. Manuscript revision: JJN, GMM, JJT, CL, GCM, TAD, EPG. Supervision: GMM. Approval of the final manuscript: JJN, GMM.

## Data Availability

The data analysed are available with permission from the Department of Health Care Access and Information (HCAI) at https://hcai.ca.gov/data-and-reports/request-data/.
